# Hyperbaric Oxygen Therapy and Female Infertility: A Systematic Review of Human Clinical Evidence

**DOI:** 10.3390/jcm15218713

**Published:** 2026-11-08

**Authors:** Enrico Moccia, Pasquale Longobardi, Vincenzo Lionetti, Sabrina Molinaro, Fabiola Troisi, Marco Scalese

**Affiliations:** 1 Divers and Special Forces Group “Teseo Tesei”, Italian Navy, 19025 Portovenere, Italy; enrico.moccia94@gmail.com; 2 Ravenna Hyperbaric Center, 48124 Ravenna, Italy; divedoc@libero.it; 3 Interdisciplinary Research Center “Health Science”, Scuola Superiore Sant’Anna, 56127 Pisa, Italy; vincenzo.lionetti@santannapisa.it; 4 Institute of Clinical Physiology, IFC, National Research Council of Italy—CNR, Via G. Moruzzi, 1, 56124 Pisa, Italy; marco.scaleseurciuoli@cnr.it; 5 Institute of Nanoscience, National Research Council of Italy—CNR, Via G. Moruzzi, 1, 56124 Pisa, Italy; fabiola.troisi@nano.cnr.it

**Keywords:** female infertility, hyperbaric oxygen therapy, HBOT, assisted reproduction, thin endometrium, poor ovarian response, sirtuins, SIRT1

## Abstract

**Background/Objectives:** Female infertility represents a major global health issue, and effective treatment remains challenging in several infertility-related conditions. Hyperbaric oxygen therapy (HBOT) has been proposed as a potential adjunctive intervention in reproductive medicine, although human clinical evidence remains limited. This systematic review aimed to evaluate the available human evidence regarding the association between HBOT and reproductive outcomes in women with infertility or infertility-related conditions. Potential biological mechanisms, including the hypothetical involvement of sirtuin-related signaling in adaptive responses to intermittent hyperoxia, were also considered. **Methods:** A systematic literature search of PubMed/MEDLINE, Scopus, LILACS and the Cochrane Library was updated through 10 May 2026. The search was restricted to studies published from 1 January 2016 to 10 May 2026, reflecting the review’s prespecified focus on approximately the preceding decade of human clinical evidence. Original human studies investigating HBOT in women with infertility or infertility-related conditions and reporting reproductive outcomes were eligible. Study selection was conducted according to predefined eligibility criteria and reported in accordance with the PRISMA 2020 statement. Methodological quality was assessed using the Newcastle–Ottawa Scale (NOS) and the certainty of evidence for the main outcomes was evaluated using the GRADE approach. Owing to substantial clinical and methodological heterogeneity and the absence of sufficiently comparable outcomes across studies, a pooled meta-analysis was not performed. **Results:** Seventy-six records were identified through electronic database searches, and 11 reports underwent full-text eligibility assessment. Two original human studies involving women with resistant thin endometrium or poor ovarian response met the eligibility criteria. In women with resistant thin endometrium undergoing frozen embryo transfer, endometrial thickness increased after HBOT (6.57 ± 1.23 vs. 5.76 ± 1.66 mm before HBOT; *p* = 0.002). Compared with propensity-score-matched concurrent controls, women receiving HBOT had a higher implantation rate (RR, 3.00; 95% CI, 1.26–7.14) and a higher intrauterine pregnancy rate after blastocyst transfer (RR, 2.96; 95% CI, 1.14–7.70). In women with poor ovarian response undergoing IVF/ICSI, fully adjusted analyses showed that HBOT was associated with a higher number of retrieved oocytes (β = 1.51; 95% CI, 0.92–2.10), available embryo rate (β = 24.29 percentage points; 95% CI, 7.27–41.31), and normal fertilization rate (β = 17.23 percentage points; 95% CI, 2.35–32.11). Clinical pregnancy occurred in 1 of 10 pre-HBOT cycles (10.0%) and 1 of 19 post-HBOT cycles (5.3%); one live birth was reported following HBOT. This single event does not provide a comparative estimate of live-birth rates. Certainty of evidence was very low for all outcomes assessed using GRADE: endometrial thickness, implantation, intrauterine pregnancy, number of oocytes retrieved, available embryo rate, and normal fertilization rate. **Conclusions:** Available human evidence reports associations between HBOT and selected reproductive outcomes in women with resistant thin endometrium or poor ovarian response. However, the evidence is based on only two small, non-randomized studies addressing different clinical populations and outcomes, and the certainty of evidence was very low for all outcomes assessed using GRADE. Current evidence is therefore insufficient to establish a causal therapeutic effect, support the routine use of HBOT for female infertility or define an optimal treatment protocol. Further well-designed prospective controlled studies are required.

## 1. Introduction

### 1.1. Rationale

Female infertility represents a major global health issue with important psychological, social and economic implications. According to the World Health Organization (WHO), approximately one in six people worldwide experience infertility during their lifetime [1]. This substantial global burden has also been highlighted in the reproductive medicine literature following the publication of WHO infertility estimates [2]. Current therapeutic strategies include pharmacological treatments, assisted reproductive technologies and interventions aimed at addressing specific causes of infertility and improving reproductive outcomes. Nevertheless, treatment outcomes remain variable, particularly in women with conditions such as poor ovarian response, impaired endometrial development or recurrent failure of assisted reproductive treatments. Hyperbaric oxygen therapy (HBOT) consists of the intermittent administration of high-concentration oxygen under increased atmospheric pressure and is currently used for several medical conditions in which increased tissue oxygen availability and modulation of oxygen-dependent biological responses may be beneficial. HBOT has been associated with multiple biological effects, including increased tissue oxygenation, modulation of oxidative stress and inflammatory responses, angiogenesis and effects on microvascular and mitochondrial function [3]. Interest has therefore emerged regarding the potential application of HBOT in reproductive medicine. Preliminary clinical studies have investigated whether HBOT may influence reproductive parameters related to endometrial development, ovarian response, fertilization, embryo development, implantation, and pregnancy. However, the human clinical evidence remains scarce and heterogeneous, and the potential therapeutic role of HBOT in female infertility has not been established.

### 1.2. Potential Biological Mechanisms

**Figure 1:**
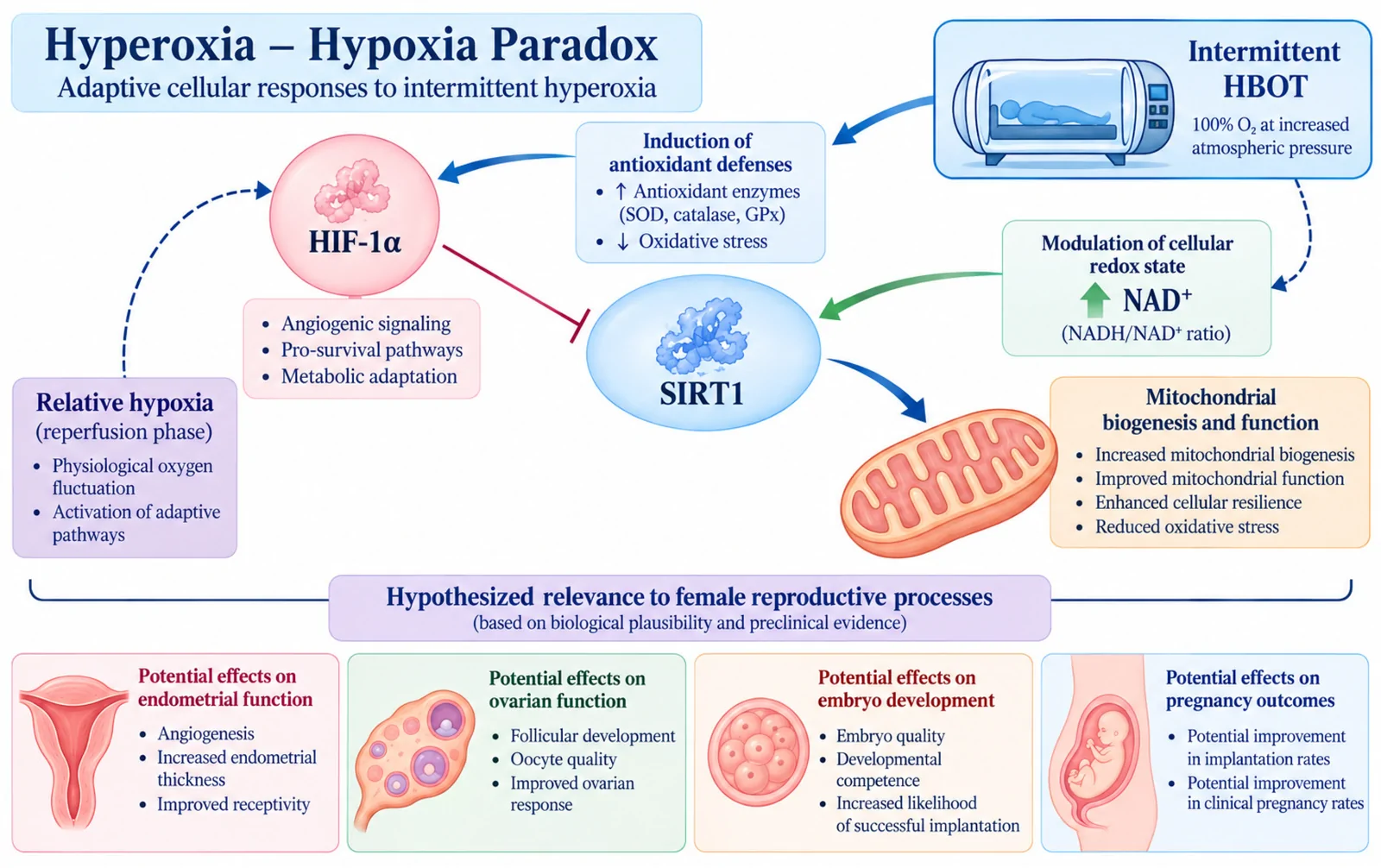
Proposed mechanistic framework linking intermittent hyperoxic exposure to adaptive cellular responses and their potential relevance to female reproductive function. Intermittent HBOT may induce adaptive responses involving antioxidant defenses, HIF-1α-related signaling, changes in cellular redox state and Nicotinamide adenine dinucleotide (NAD^+^) availability and SIRT1-related pathways, potentially influencing mitochondrial biogenesis and function [3,4,5,6,7,8]. These mechanisms could provide biological hypotheses linking HBOT to endometrial, ovarian and embryo-related processes; however, their involvement in female reproductive outcomes remains hypothetical and has not been directly investigated in the human clinical studies included in this review [9,10]. The figure was generated by the authors with the assistance of OpenAI’s ChatGPT image-generation tool and subsequently reviewed for scientific content.

One proposed theoretical framework underlying the possible effects of HBOT on reproductive function involves modulation of oxidative stress and adaptive cellular responses to intermittent hyperoxia. In particular, the “hyperoxic–hypoxic paradox” (Figure 1) describes the phenomenon whereby repeated intermittent hyperoxic exposures may trigger adaptive molecular responses that overlap with pathways typically activated by hypoxia [3]. Among the molecular pathways potentially involved, sirtuin-related signaling is of particular interest because sirtuins participate in the regulation of oxidative stress, mitochondrial function, inflammation, apoptosis and cellular metabolism [4,5]. Sirtuin 1 (SIRT1) has been implicated in several processes related to reproductive physiology, including ovarian function, granulosa-cell homeostasis and cellular responses to oxidative stress [6,7]. Moreover, the mitochondrial Sirtuin 3 (SIRT3) has been associated with protection against oxidative stress and maintenance of cellular homeostasis in oocytes and early embryos [8]. Nevertheless, the relationship between HBOT, sirtuin modulation, and female reproductive outcomes remains largely theoretical. Direct clinical evidence demonstrating that HBOT improves reproductive outcomes through modulation of SIRT1, SIRT3, or related pathways is currently lacking. These mechanisms should therefore be regarded as biologically plausible hypotheses rather than established therapeutic mechanisms in female infertility.

### 1.3. Clinical Evidence and Applications Investigated

The available human literature has explored HBOT in different infertility-related clinical settings. In women with resistant thin endometrium undergoing frozen embryo transfer, HBOT has been investigated in relation to endometrial development, embryo implantation and pregnancy outcomes [9]. More recently, HBOT has also been evaluated in women with poor ovarian response undergoing In Vitro Fertilisation (IVF)/Intracytoplasmic Sperm Injection (ICSI), with particular attention to ovarian response, oocyte retrieval, fertilization and embryo-related outcomes [10]. These studies address distinct components of reproductive function and use different study designs, comparator structures and HBOT protocols. Consequently, the available clinical evidence cannot currently be interpreted as demonstrating a uniform therapeutic effect of HBOT across female infertility. Furthermore, differences in treatment pressure, session duration, number of sessions, timing of HBOT relative to assisted reproductive treatment and outcome definitions limit direct comparisons between studies.

### 1.4. Why It Is Important to Conduct This Review

Despite the biological plausibility of oxygen-dependent effects on reproductive tissues, the clinical evidence regarding HBOT in female infertility remains limited and fragmented. Importantly, experimental evidence and observations in animal models cannot be directly extrapolated to clinical efficacy in women. An updated systematic evaluation restricted to original human studies is therefore needed to distinguish the available clinical evidence from experimental and mechanistic hypotheses. A systematic synthesis of the available human evidence may help clarify the reproductive outcomes that have been investigated, assess the methodological quality and certainty of the evidence, identify sources of clinical and methodological heterogeneity and determine the major gaps that should be addressed by future research. Such an assessment is particularly relevant before HBOT can be considered for broader investigation or potential application in reproductive medicine.

### 1.5. Objectives

The primary objective of this systematic review was to evaluate human clinical evidence published from 1 January 2016 to 10 May 2026 regarding the potential association between hyperbaric oxygen therapy and reproductive outcomes in women with infertility or infertility-related conditions. Specifically, the review aimed to evaluate reported effects or associations involving endometrial development, embryo implantation, pregnancy, ovarian response, oocyte retrieval, fertilization and embryo-related outcomes. Secondary objectives were to characterize the HBOT protocols and methodological features of the available studies, assess their methodological quality and the certainty of evidence and identify the principal sources of clinical and methodological heterogeneity. In addition, this review discusses potential biological mechanisms that may provide a rationale for further investigation of HBOT in reproductive medicine, while clearly distinguishing mechanistic hypotheses from demonstrated clinical effects.

## 2. Materials and Methods

### 2.1. Eligibility Criteria

Studies were considered eligible if they investigated the use of hyperbaric oxygen therapy (HBOT) in women with infertility or infertility-related conditions and reported reproductive or clinically relevant fertility-related outcomes. Only original human studies were eligible for inclusion. Studies published from 1 January 2016 to 10 May 2026 were eligible. This publication period was prespecified because the review aimed to examine human clinical evidence from approximately the preceding decade. No language restrictions were applied. Studies were excluded if they: (1) were conducted exclusively in animals or in vitro models; (2) investigated male infertility without reporting separate data for women; (3) did not evaluate infertility or an infertility-related condition or outcome; (4) did not investigate HBOT as the intervention of interest; or (5) represented reviews, systematic reviews, meta-analyses, book chapters, editorials, commentaries, or other forms of secondary literature without original patient data. Reports for which the full text could not be retrieved were not included in the eligibility assessment. Eligibility criteria were structured according to the Population, Intervention, Comparator, Outcomes and Study design (PICOS) framework. Population: Women with infertility, undergoing assisted reproductive treatment, or presenting with infertility-related conditions affecting reproductive outcomes. Intervention: Hyperbaric oxygen therapy (HBOT), administered as the main intervention or as an adjunct to standard infertility or assisted reproductive treatment. Comparator: Standard treatment, no HBOT, a concurrent control group or the patients’ pre-HBOT condition in pre–post studies. Outcomes: Reproductive and fertility-related outcomes, including clinical pregnancy, embryo implantation, endometrial thickness, oocyte yield, fertilization, embryo availability or quality and other clinically relevant reproductive outcomes reported by the included studies. Study design: Original human clinical studies reporting primary data, including observational cohort studies and non-randomized interventional studies. The review protocol was not prospectively registered in PROSPERO or another public repository.

### 2.2. Information Sources

A literature search was conducted in PubMed/MEDLINE, Scopus, LILACS, and the Cochrane Library. The search was updated through 10 May 2026. Reference lists of relevant articles were also examined to identify additional potentially eligible studies. The grey literature, clinical trial registries, and conference abstracts were not searched.

### 2.3. Search Strategy

Database-specific search strategies were developed using combinations of terms related to female infertility, assisted reproduction, reproductive outcomes and hyperbaric oxygen therapy. Search syntax was adapted to the indexing system and search functionalities of each database. Where applicable, database-specific field restrictions and publication-date filters were applied. The search was restricted to publications from 1 January 2016 to 10 May 2026, in accordance with the prespecified publication-date eligibility criterion described in Section 2.1. The complete electronic search strategies, including the exact search strings, field restrictions and filters applied, are reported in the Supplementary Materials. Potentially eligible records were screened according to the predefined eligibility criteria.

### 2.4. Selection Process

All records identified through the database searches were collected and duplicates were removed before screening. Study selection was performed independently by two authors. Titles and abstracts were independently screened against the predefined eligibility criteria and potentially relevant reports were subsequently sought for full-text retrieval. Full-text articles were independently assessed for eligibility by the same two reviewers. Disagreements at any stage of the selection process were resolved through discussion and consensus. Reports for which the full texts could not be downloaded were recorded as not retrieved in the PRISMA flow diagram and were not assessed for eligibility. Reasons for exclusion at the full-text stage were recorded. The study selection process was documented using the PRISMA 2020 flow diagram.

### 2.5. Data Extraction and Management

Data extraction was performed independently by two authors using a standardized data-extraction form. Discrepancies were resolved through discussion and consensus. The following information was collected, where available: first author, year of publication, country, study design, study population, sample size, infertility-related condition, characteristics of the HBOT intervention, comparator, reproductive treatment setting, outcomes assessed and main findings. For HBOT protocols, pressure expressed in atmospheres absolute (ATA), session duration, oxygen exposure, number of treatment sessions and timing of HBOT in relation to the reproductive treatment were extracted when reported. For continuous outcomes, means and standard deviations or other available measures of effect were extracted. For dichotomous outcomes, the numbers of events and participants in each group were extracted when available. Missing or insufficiently reported data were not imputed.

### 2.6. Outcomes and Effect Measures

The outcomes of interest were reproductive and fertility-related outcomes reported by the eligible studies. These included endometrial thickness, implantation rate, clinical pregnancy, intrauterine pregnancy, live birth, number of oocytes retrieved, fertilization outcomes and embryo availability and quality. Additional reproductive outcomes reported by the included studies were extracted when relevant. Because the included studies investigated different infertility-related conditions and different stages of assisted reproductive treatment, outcomes were synthesized according to their clinical definition and the population in which they were assessed. Live-birth findings were summarized descriptively because the included studies did not provide comparative live-birth estimates. For dichotomous outcomes, risk ratios (RRs) with 95% confidence intervals (CIs) were calculated from the reported event counts when sufficient data were available. For continuous outcomes, mean differences (MDs) were reported when directly available or calculable from the published data. When included studies reported adjusted regression estimates, these were retained in their original form, together with the corresponding 95% CIs, rather than being treated as unadjusted mean differences. Where relevant, the original outcome data and statistical significance reported by the individual studies were also presented.

### 2.7. Risk of Bias and Methodological Quality Assessment

The methodological quality of the included non-randomized studies was assessed using the Newcastle–Ottawa Scale (NOS) [11]. The NOS evaluates three domains: selection of study groups, comparability of groups, and ascertainment of exposure or outcome, with a maximum score of nine stars. Total and domain scores were reported descriptively and were not interpreted as stand-alone measures of overall risk of bias. Study-specific design limitations not fully captured by the NOS score were considered in the GRADE assessment for each outcome, including selection, residual confounding, and the presence or absence of an independent concurrent comparator. Detailed domain-level NOS assessments are provided in Supplementary Table S3. Two authors independently performed the NOS assessments, and disagreements were resolved through discussion and consensus. The completed PRISMA 2020 checklist is provided in Supplementary Table S4.

### 2.8. Synthesis Methods

Owing to substantial clinical and methodological heterogeneity between the included studies, a pooled meta-analysis was not performed. The studies differed in the infertility-related condition investigated, the assisted reproductive treatment setting, the HBOT protocol, the comparator structure, and the reproductive outcomes assessed. Chen et al. [9] investigated women with resistant thin endometrium undergoing frozen embryo transfer using a concurrent propensity-score-matched comparison group, whereas Meng et al. [10] evaluated women with poor ovarian response using a pre–post cohort design. No principal outcome was reported by more than one sufficiently comparable study to support a clinically meaningful pooled effect estimate. Results were therefore synthesized narratively and quantitatively using study-specific effect estimates where sufficient data were available. For dichotomous outcomes, RRs with 95% CIs were calculated from the reported numbers of events and participants when appropriate. For continuous outcomes, MDs were reported when directly available or calculable, whereas adjusted regression coefficients were retained when these represented the principal effect estimates reported by the original study. No statistical pooling was undertaken when outcomes, populations, or comparator structures were not sufficiently comparable. Statistical significance was interpreted using a two-sided threshold of *p* < 0.05 where reported by the original studies.

### 2.9. Assessment of Reporting Bias

Given the small number of eligible studies and the fact that each principal outcome was supported by only one study, formal assessment of publication bias or small-study effects using funnel plots or statistical tests was not considered appropriate. The possibility of publication and selective reporting bias could therefore not be excluded.

### 2.10. Reporting Guideline

This systematic review was conducted and reported in accordance with the Preferred Reporting Items for Systematic Reviews and Meta-Analyses (PRISMA) 2020 statement [12].

## 3. Results

### 3.1. Study Selection

**Figure 2:**
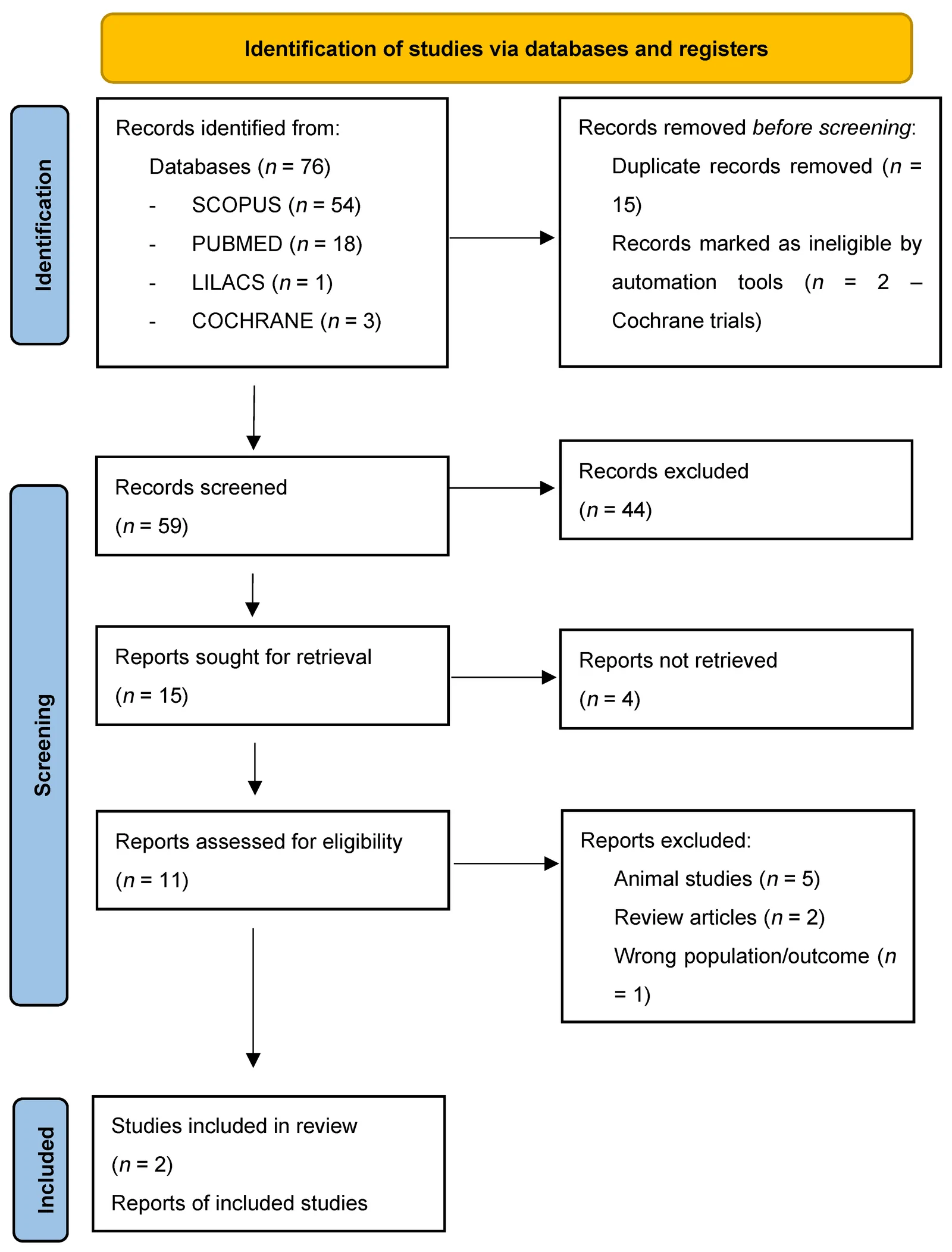
PRISMA 2020 flow diagram of study selection [12].

The updated literature search identified 76 records across the electronic databases: 54 from Scopus, 18 from PubMed/MEDLINE, 1 from LILACS and 3 from the Cochrane Library. After removal of 15 duplicate records and 2 records identified as ineligible before screening, 59 records underwent title and abstract screening. Of these, 44 records were excluded because they did not meet the predefined eligibility criteria. The full texts of 15 potentially eligible reports were sought; 4 could not be downloaded and were therefore not assessed for eligibility. The remaining 11 reports underwent full-text eligibility assessment. Following full-text assessment, 9 reports were excluded: 5 animal studies, 2 review articles without original patient data, 1 study investigating an ineligible population or outcome and 1 book chapter representing secondary literature without original clinical data. Ultimately, 2 original human studies met the eligibility criteria and were included in the systematic review: Chen et al. (2023) [9] and Meng et al. (2025) [10]. The study selection process and reasons for exclusion at the full-text stage are presented in the PRISMA 2020 flow diagram (Figure 2). Details of reports excluded following full-text assessment and the corresponding reasons for exclusion are provided in Supplementary Table S2.

### 3.2. Study Characteristics

Two studies, published in 2023 and 2025, met the eligibility criteria. Both were conducted in China and investigated HBOT as an adjunctive intervention in women undergoing assisted reproductive treatment, although they addressed distinct infertility-related conditions and different stages of the reproductive treatment pathway. Chen et al. (2023) [9] investigated HBOT in women with resistant thin endometrium undergoing frozen embryo transfer. The study included 41 women receiving HBOT and used a propensity-score-matched concurrent control group. The main outcomes included endometrial thickness, cycle cancellation, implantation rate, and intrauterine pregnancy rate. Meng et al. (2025) [10] evaluated 41 women with poor ovarian response undergoing IVF/ICSI treatment using a pre–post cohort design. Reproductive outcomes obtained during treatment cycles before HBOT were compared with those obtained after HBOT. Outcomes included the number of oocytes retrieved, normal fertilization rate, available embryo rate and other measures of ovarian response and embryo development. The HBOT protocols differed between studies with respect to treatment pressure, session duration, number of sessions, and timing of treatment in relation to assisted reproduction. Detailed characteristics of the included studies and HBOT protocols are reported in Table 1.

### 3.3. Risk of Bias in Included Studies

The methodological quality of the two included studies was assessed using the Newcastle–Ottawa Scale (NOS). Chen et al. (2023) [9] scored 9/9 stars, while Meng et al. (2025) [10] scored 7/9 stars (Supplementary Table S3). These scores are reported descriptively and should not be interpreted as indicating the absence of important risk-of-bias concerns. In Chen et al., the concurrent comparison group and propensity-score matching addressed measured factors, but treatment allocation was not randomized and residual confounding cannot be excluded. In Meng et al., the pre–post design compared cycles in the same patients without an independent concurrent control group. Although adjusted analyses accounted for selected measured covariates, temporal effects, regression to the mean, and residual confounding remain possible. These design limitations were considered separately in the GRADE assessment for each outcome.

### 3.4. Results of Individual Studies

#### 3.4.1. Resistant Thin Endometrium

Chen et al. (2023) [9] evaluated HBOT in women with resistant thin endometrium undergoing frozen embryo transfer using both within-patient pre–post analyses and a propensity-score-matched concurrent control comparison. Endometrial thickness increased after HBOT compared with the patients’ pre-HBOT treatment cycles (6.57 ± 1.23 vs. 5.76 ± 1.66 mm; *p* = 0.002). Cycle cancellation also decreased in the within-patient comparison after HBOT. Following propensity-score matching, 41 women receiving HBOT were compared with 41 concurrent controls receiving standard treatment without HBOT. In this comparison, the implantation rate was 33.3% (14/42) in the HBOT group and 11.1% (6/54) in the control group, corresponding to an RR of 3.00 (95% CI, 1.26–7.14). Among women undergoing blastocyst transfer, the intrauterine pregnancy rate was 53.8% (14/26) in the HBOT group and 18.2% (4/22) in the control group (RR, 2.96; 95% CI, 1.14–7.70). In contrast, cycle cancellation did not significantly differ between the HBOT and matched control groups. These findings suggest favorable within-patient changes in endometrial development after HBOT and higher implantation and intrauterine pregnancy rates compared with matched controls. However, given the non-randomized design and limited sample size, these findings should be interpreted as associations rather than evidence of a causal treatment effect.

#### 3.4.2. Poor Ovarian Response

Meng et al. (2025) [10] investigated HBOT in 41 women with poor ovarian response undergoing IVF/ICSI using a self-controlled pre–post cohort design. Outcomes from treatment cycles performed after HBOT were compared with those obtained in the same women before HBOT. In the fully adjusted generalized estimating equation model, HBOT was associated with an increase in the number of retrieved oocytes (β = 1.51; 95% CI, 0.92–2.10), available embryo rate (β = 24.29 percentage points; 95% CI, 7.27–41.31), and normal fertilization rate (β = 17.23 percentage points; 95% CI, 2.35–32.11). The model accounted for age, anti-Müllerian hormone, antral follicle count, body mass index, controlled ovarian stimulation protocol, and trigger method. Exploratory dose–response analyses suggested that the most favorable improvements were observed with 4–7 HBOT sessions. Clinical pregnancy occurred in 1 of 10 pre-HBOT cycles (10.0%) and 1 of 19 post-HBOT cycles (5.3%). The pregnancy achieved using a pre-HBOT cycle ended in early miscarriage, whereas the pregnancy following HBOT resulted in a vaginal delivery of a healthy male infant at 40 + 2 weeks, with no maternal or fetal complications reported. This single live birth does not provide a comparative live-birth estimate. Overall, post-HBOT cycles showed more favorable ovarian response, fertilization, and embryo-related outcomes than the corresponding pre-HBOT cycles. However, because the study used a self-controlled pre–post design without an independent concurrent control group, temporal effects, regression to the mean, and residual confounding cannot be excluded, and these findings cannot establish a causal effect of HBOT.

### 3.5. Results of Syntheses

A pooled meta-analysis was not performed because no outcome was reported by more than one sufficiently comparable study. The two included studies addressed different clinical populations, different stages of assisted reproductive treatment, different HBOT protocols and different comparator structures. Chen et al. [9] evaluated resistant thin endometrium using a concurrent propensity-score-matched control group, whereas Meng et al. [10] evaluated poor ovarian response using within-patient comparisons between pre- and post-HBOT cycles. Pooling these estimates into a single summary effect would therefore not provide a clinically meaningful estimate of HBOT efficacy. Accordingly, the available evidence was synthesized narratively, with study-specific quantitative effect estimates reported for individual outcomes where sufficient data were available.

### 3.6. Reporting Biases

Given that only two studies met the eligibility criteria and each principal outcome was supported by a single study, formal assessment of publication bias or small-study effects using funnel plots or statistical tests was not considered appropriate. The possibility of reporting and publication bias therefore cannot be excluded.

### 3.7. Certainty of Evidence

Two authors independently assessed the certainty of evidence using the Grading of Recommendations Assessment, Development and Evaluation (GRADE) approach [13], resolving disagreements through discussion and consensus. They considered risk of bias, inconsistency, indirectness, imprecision, and other considerations, with risk-of-bias judgments informed by the Newcastle–Ottawa Scale assessment. Evidence from the included non-randomized studies started at low certainty and was downgraded where warranted. The final certainty was very low for all six outcomes: intrauterine pregnancy after blastocyst transfer, implantation rate, endometrial thickness, number of oocytes retrieved, available embryo rate, and normal fertilization rate. Serious risk-of-bias concerns led to downgrading across all outcomes, and the outcomes evaluated by Meng et al. [10] were further downgraded for serious imprecision due to the limited information size. Each outcome was supported by a single study, so consistency across independent studies could not be assessed. The full GRADE assessment and effect estimates are reported in the Summary of Findings table (Table 2), generated using GRADEpro GDT (McMaster University and Evidence Prime) [14].

## 4. Discussion

This systematic review provides an updated assessment of the available human evidence regarding the potential role of hyperbaric oxygen therapy (HBOT) in female infertility and infertility-related conditions. Despite a comprehensive literature search, only two original human studies met the predefined eligibility criteria, highlighting the scarcity of clinical evidence in this field. The studies addressed different clinical conditions and stages of assisted reproduction: Chen et al. (2023) investigated women with resistant thin endometrium undergoing frozen embryo transfer, whereas Meng et al. (2025) evaluated women with poor ovarian response undergoing IVF/ICSI [9,10]. In women with resistant thin endometrium, Chen et al. reported increased endometrial thickness after HBOT compared with the patients’ pre-HBOT cycles, while higher implantation and intrauterine pregnancy rates were observed in comparisons with propensity-score-matched concurrent controls. In women with poor ovarian response, Meng et al. reported more favorable post-HBOT outcomes than in the patients’ pre-HBOT treatment cycles, with associations persisting for several outcomes in adjusted generalized estimating equation analyses [10]. Meng et al. reported clinical pregnancy in 1 of 10 pre-HBOT cycles (10.0%) and 1 of 19 post-HBOT cycles (5.3%), and one live birth following HBOT. This single event does not provide a comparative estimate of live-birth rates. Taken together, the findings suggest possible associations between HBOT and selected reproductive outcomes in specific infertility-related conditions, but they do not establish a causal therapeutic effect or support routine clinical use. The two studies should not be interpreted as showing a single, uniform effect of HBOT on female infertility. They investigated distinct clinical conditions and different components of the reproductive process. Chen et al. primarily addressed an endometrial factor, whereas Meng et al. investigated ovarian response and subsequent embryological outcomes. The comparator structures also differed: Chen et al. used a propensity-score-matched concurrent comparison group, whereas Meng et al. used a self-controlled pre–post design [9,10]. These differences, together with variation in HBOT pressure, treatment duration, number of sessions, and timing relative to assisted reproductive treatment, precluded a clinically meaningful pooled meta-analysis. The certainty of the evidence was very low across the assessed outcomes after consideration of risk of bias. Although Chen et al. and Meng et al. received Newcastle–Ottawa Scale scores of 9/9 and 7/9, respectively, these scores should be interpreted alongside the design-specific limitations of each study. The NOS scores do not eliminate concerns about residual confounding in Chen et al. or the absence of an independent concurrent control group in Meng et al. Both studies were non-randomized and involved relatively small samples, and each outcome was supported by only one study. Independent replication is lacking, and residual confounding cannot be excluded. Intermittent hyperoxia has been proposed to trigger adaptive cellular responses, potentially involving redox regulation and sirtuin-related signaling [3,4,5,6,7,8]. However, neither included clinical study measured these pathways or assessed whether they mediated the reported reproductive outcomes. These mechanisms therefore remain hypotheses and cannot explain the clinical associations observed in this review. Translational studies are needed to determine whether these pathways are relevant to reproductive outcomes. Several experimental studies identified during the updated screening investigated reproductive effects of hyperbaric oxygen exposure in animal models. These studies were excluded from the clinical evidence synthesis because the eligibility criteria were restricted to original human studies. Experimental evidence may contribute to understanding potential mechanisms and generating hypotheses, but it cannot establish clinical efficacy in women. This distinction is particularly important in a field where eligible human evidence remains extremely limited. The potential use of hyperbaric oxygen-based interventions in reproductive medicine predates the two clinical studies included in this review. Earlier preliminary reports investigated anti-Müllerian hormone levels, endometrial characteristics, ovarian stimulation, and reproductive outcomes in selected infertility populations while other publications discussed HBOT or related approaches more broadly in the context of infertility [15,16,17,18,19,20]. HBOT has also been discussed or investigated in other gynecological settings, including pelvic inflammatory disease and ovarian function following ovarian cystectomy [21,22]. These publications provide historical and hypothesis-generating context. They were not included in the present synthesis because they fell outside the prespecified publication period or did not meet one or more of the eligibility criteria, including those concerning study design, population, or outcomes. Their exclusion reflects the scope of this review and does not imply that no earlier clinical reports exist. The available evidence remains preliminary and hypothesis-generating. It provides a rationale for further investigation, not a basis for recommending HBOT as an established treatment for female infertility. Future studies should determine whether the reported associations are reproducible, identify the clinical phenotypes most likely to benefit, and establish whether any potential effect translates into clinically meaningful outcomes, particularly live birth. HBOT protocols should be reported in sufficient detail, including chamber type, treatment pressure, oxygen-exposure duration, session frequency, total number of sessions, and timing relative to assisted reproductive treatment. Future studies should also systematically assess adverse events, tolerability, and treatment burden. Finally, the review protocol was not prospectively registered in PROSPERO or another public repository. Although the eligibility criteria and study-selection procedures are described in the Methods, the absence of a publicly accessible protocol limits independent verification of the planned methods and should be considered when interpreting the findings.

### 4.1. Strengths and Limitations

A strength of this systematic review is the updated literature search and the restriction of the clinical evidence synthesis to original human studies. This approach avoids combining experimental animal evidence with clinical outcomes and allows a clearer assessment of the current evidence directly applicable to women with infertility or infertility-related conditions. Study-level methodological quality was assessed using the Newcastle–Ottawa Scale, while certainty of evidence was evaluated separately using the GRADE framework. Several important limitations should nevertheless be acknowledged. First, only two original human studies fulfilled the eligibility criteria, and both were conducted in China, limiting the geographical diversity and potentially the generalizability of the findings [9,10]. Second, both studies were non-randomized and included relatively small samples. Residual confounding, selection bias, temporal changes in treatment response, and other unmeasured factors therefore cannot be excluded. Third, substantial clinical and methodological heterogeneity was present. The studies investigated different infertility-related conditions, used different comparator structures, applied different HBOT protocols, and evaluated different reproductive outcomes. Consequently, no principal outcome was independently evaluated by more than one sufficiently comparable study, and quantitative pooling was considered inappropriate. This also prevented meaningful statistical assessment of between-study heterogeneity and publication bias. Grey literature, clinical trial registries, and conference abstracts were not searched; therefore, unpublished or ongoing studies may have been missed. The full texts of four reports could not be downloaded, so their eligibility and findings could not be assessed. The possibility that eligible evidence was missed cannot therefore be excluded, and this should be considered when interpreting the completeness of the review. Another important limitation concerns the clinical relevance of the available endpoints. Several outcomes evaluated in the included studies, including endometrial thickness, oocyte yield, fertilization rate, and embryo-related parameters, represent intermediate reproductive outcomes rather than definitive measures of reproductive success. Pregnancy-related outcomes were reported in both studies. Chen et al. [9] reported implantation and intrauterine pregnancy rates, whereas Meng et al. [10] reported clinical pregnancy in 1 of 10 pre-HBOT cycles (10.0%) and 1 of 19 post-HBOT cycles (5.3%), and one live birth following HBOT. However, this single event does not provide a comparative live-birth estimate. Therefore, improvements in intermediate reproductive parameters should not be assumed to translate into improved live-birth rates. Safety reporting was limited in the included studies. Chen et al. reported no HBOT-related complications, such as headache or discomfort, whereas Meng et al. described physician monitoring for adverse reactions but did not provide a dedicated summary of adverse-event outcomes [9,10]. Therefore, the available reports do not establish the safety or tolerability of HBOT in women undergoing reproductive treatment. Treatment burden also warrants consideration: Chen et al. reported a mean of 28.48 ± 15.24 HBOT treatments, with each session lasting 100 min, whereas Meng et al. reported a mean of 4.46 ± 2.26 sessions lasting 120 min [9,10]. Future studies should prospectively report adverse events, tolerability, treatment discontinuations, and treatment burden alongside reproductive outcomes.

Fourth, the available evidence does not establish an optimal HBOT protocol for reproductive indications. Treatment pressure, session duration, total number of sessions and timing relative to ovarian stimulation or embryo transfer differed between the included studies [9,10]. Therefore, the current findings cannot be used to define a standardized therapeutic regimen. Finally, although several biological mechanisms may plausibly connect hyperbaric oxygen exposure with reproductive physiology, these mechanisms were not directly investigated by the included human studies. Mechanistic interpretations should therefore remain exploratory. The review was intentionally restricted to studies published from 1 January 2016 to 10 May 2026. Earlier clinical reports were therefore outside its prespecified scope, even where they may have addressed related populations or outcomes. The findings should be interpreted as a synthesis of human clinical evidence published during approximately the preceding decade, rather than as a comprehensive historical review.

### 4.2. Evidence Gaps and Implications for Future Research

The main gaps in the current evidence are the small number of non-randomized human studies, the lack of independent replication, the absence of comparative live-birth estimates, limited reporting of safety and treatment burden, and the heterogeneity of the populations, HBOT protocols, and outcomes studied. The proposed biological mechanisms have also not been directly evaluated in the included clinical studies. Further well-designed prospective human studies are required before the role of HBOT in female infertility can be established. Randomized controlled trials would provide more robust evidence, although carefully designed prospective controlled cohort studies could also contribute important preliminary data. Future studies should clearly define the infertility phenotype and avoid combining clinically distinct populations. Resistant thin endometrium, poor ovarian response, recurrent implantation failure and other infertility-related conditions should be investigated separately unless a clear biological and clinical rationale supports their combined analysis.

HBOT protocols should be standardized and reported in sufficient detail, including chamber type, treatment pressure in atmospheres absolute (ATA), duration of oxygen exposure, session duration, treatment frequency, total number of sessions, and timing relative to ovarian stimulation, endometrial preparation, ovulation triggering, or embryo transfer. Future investigations should prioritize clinically relevant reproductive endpoints, including clinical pregnancy and, particularly, live birth, while also assessing implantation, miscarriage, ovarian response, oocyte yield, fertilization, embryo development and endometrial parameters where appropriate. Future studies should prospectively report adverse events, tolerability, treatment discontinuations and treatment burden. Finally, translational studies integrating clinical reproductive outcomes with biomarkers of tissue oxygenation, angiogenesis, inflammation, oxidative stress, and sirtuin-related signaling could help determine whether the biological mechanisms proposed for HBOT are actually involved in reproductive responses. Such studies would help bridge the current gap between experimental plausibility and clinical evidence.

If a sufficiently large and clinically comparable evidence base becomes available in future updates, subgroup analyses according to infertility phenotype and HBOT protocol characteristics may become informative. Meta-regression could also be considered to explore potential sources of heterogeneity; however, the current number of eligible studies is far too small to support such analyses.

## 5. Conclusions

The available human evidence on hyperbaric oxygen therapy in female infertility remains limited. This systematic review identified only two eligible original clinical studies, investigating distinct infertility-related conditions and different stages of assisted reproductive treatment.

In women with resistant thin endometrium undergoing frozen embryo transfer, endometrial thickness increased following HBOT compared with pre-HBOT cycles, while higher implantation and intrauterine pregnancy rates were observed compared with propensity-score-matched concurrent controls. In women with poor ovarian response undergoing IVF/ICSI, post-HBOT cycles were associated with a higher number of retrieved oocytes and more favorable fertilization and embryo-related outcomes compared with pre-HBOT cycles. Accordingly, the current evidence should be considered preliminary and hypothesis-generating. The certainty of evidence was very low for all outcomes assessed using GRADE, and no causal relationship between HBOT and improved reproductive outcomes can currently be established. The available data are therefore insufficient to support HBOT as a routine treatment for female infertility or to define an optimal therapeutic protocol. The associations observed in the available human studies, together with the biological plausibility of oxygen-dependent effects on reproductive tissues, support the need for further investigation. Well-designed prospective controlled studies, preferably randomized clinical trials, are needed to confirm these findings, identify the infertility phenotypes most likely to benefit, standardize HBOT protocols, assess safety, and determine whether potential improvements in intermediate reproductive outcomes translate into clinically meaningful endpoints, particularly live birth.

## Figures and Tables

**Table 1:** Characteristics and main findings of the studies included in the review. The table summarizes study design, population and infertility-related condition, HBOT protocol, comparator, outcomes assessed, and key findings. Abbreviations: ATA, atmospheres absolute; CI, confidence interval; GEE, generalized estimating equation; HBOT, hyperbaric oxygen therapy; IVF/ICSI, in vitro fertilization/intracytoplasmic sperm injection.

Study, Country, and Design	Population, Condition, and Sample	HBOT Protocol	Comparator	Outcomes and Main Findings
Chen et al., 2023 [9] China Prospective cohort study with a pre–post HBOT analysis and a propensity-score-matched concurrent control comparison.	Women undergoing frozen embryo transfer with resistant thin endometrium (<7 mm) unresponsive to conventional therapies; predominantly associated with intrauterine adhesions. 41 women receiving HBOT and 41 matched controls.	Multiplace chamber; 2.5 ATA; 100 min/session, including 70 min of 100% oxygen; at least 10 sessions during the proliferative phase before endometrial transformation.	Concurrent matched control group receiving standard endometrial preparation without HBOT.	Outcomes: endometrial thickness, cycle cancellation, implantation, intrauterine pregnancy, biochemical pregnancy, and miscarriage. Endometrial thickness increased after HBOT compared with the pre-HBOT cycle (6.57 ± 1.23 vs. 5.76 ± 1.66 mm; *p* = 0.002). Compared with matched controls, the HBOT group had higher implantation rates (33.3% vs. 11.1%; *p* = 0.008) and intrauterine pregnancy rates after blastocyst transfer (53.8% vs. 18.2%; *p* = 0.017).
Meng et al., 2025 [10] China Self-controlled pre–post cohort study without an independent concurrent control group.	Women aged 20–45 years with poor ovarian response according to Bologna criteria undergoing IVF/ICSI. 41 women, with one pre-HBOT and one post-HBOT cycle per woman; 13 additional second post-HBOT cycles were included in exploratory analyses.	Multiplace chamber; 2.0 ATA; 120 min/session; HBOT began on day 3 of menstruation and continued daily or every other day until trigger day; mean 4.46 ± 2.26 sessions.	The same women’s pre-HBOT treatment cycles.	Outcomes: oocyte retrieval, follicle output rate, follicle-to-oocyte index, normal fertilization, available embryo rate, embryo quality, and pregnancy outcomes. In adjusted GEE analyses, HBOT was associated with more retrieved oocytes (β = 1.51; 95% CI, 0.92–2.10), a higher available embryo rate (β = 24.29 percentage points; 95% CI, 7.27–41.31), and a higher normal fertilization rate (β = 17.23 percentage points; 95% CI, 2.35–32.11). Clinical pregnancy rates were 10.0% (1/10) before HBOT and 5.3% (1/19) after HBOT. One live birth was reported following HBOT; this single event does not provide a comparative estimate of live-birth rates.

**Table 2:** GRADE assessment of the certainty of evidence for hyperbaric oxygen therapy (HBOT) compared with standard treatment, no HBOT, or the pre-treatment condition in women with infertility or infertility-related conditions undergoing assisted reproductive treatment. Setting: Assisted reproductive medicine and infertility treatment. Abbreviations: CI, confidence interval; HBOT, hyperbaric oxygen therapy; MD, mean difference; RR, risk ratio; β, adjusted regression coefficient. Explanations: ^a^ Downgraded one level for serious risk of bias because both studies were non-randomized. In Chen et al., propensity-score matching could address only measured confounders, and the endometrial-thickness estimate was based on a pre–post comparison. In Meng et al., the self-controlled pre–post design lacked an independent concurrent control group; temporal effects, regression to the mean, and residual confounding could not be excluded. ^b^ Downgraded one level for serious imprecision because the estimates were based on a single small study with a limited sample size (*n* = 41), resulting in insufficient information size.

Outcome	Participants/Observations	Relative Effect (95% CI)	Absolute Effect	GRADE Assessment	Certainty	Importance
Intrauterine pregnancy after blastocyst transfer, confirmed by ultrasound	1 study [9]; 26 HBOT and 22 control participants; events: 14/26 vs. 4/22	RR 2.96 (1.14–7.70)	356 more per 1000 (from 25 more to 1000+ more)	Risk of bias: serious ^a^; inconsistency: not serious; indirectness: not serious; imprecision: not serious; other considerations: none	Very low ^a^	Critical
Implantation rate	1 study [9]; 41 HBOT and 41 control participants; 14/42 vs. 6/54 embryos implanted	RR 3.00 (1.26–7.14)	222 more per 1000 (from 29 more to 682 more)	Risk of bias: serious ^a^; inconsistency: not serious; indirectness: not serious; imprecision: not serious; other considerations: none	Very low ^a^	Critical
Endometrial thickness, measured by transvaginal ultrasound	1 study [9]; 41 women; pre-HBOT and post-HBOT measurements	Not reported	MD 0.81 mm higher	Risk of bias: serious ^a^; inconsistency: not serious; indirectness: not serious; imprecision: not serious; other considerations: none	Very low ^a^	Important
Number of oocytes retrieved per stimulation cycle	1 study [10]; 41 women, paired pre–post cycles	Not reported	Adjusted β = 1.51 oocytes (95% CI, 0.92–2.10)	Risk of bias: serious ^a^; inconsistency: not serious; indirectness: not serious; imprecision: serious ^b^; other considerations: none	Very low ^ab^	Critical
Available embryo rate, percentage of available embryos among fertilized oocytes	1 study [10]; 41 women, paired pre–post cycles	Not reported	Adjusted β = 24.29 percentage points (95% CI, 7.27–41.31)	Risk of bias: serious ^a^; inconsistency: not serious; indirectness: not serious; imprecision: serious ^b^; other considerations: none	Very low ^ab^	Important
Normal fertilization rate, percentage of normally fertilized oocytes (2PN)	1 study [10]; 41 women, paired pre–post cycles	Not reported	Adjusted β = 17.23 percentage points (95% CI, 2.35–32.11)	Risk of bias: serious ^a^; inconsistency: not serious; indirectness: not serious; imprecision: serious ^b^; other considerations: none	Very low ^ab^	Critical

## Data Availability

No new data were created or analyzed in this study. All data supporting the findings of this systematic review are derived from the published studies cited in the article and the accompanying Supplementary Materials.

## Supplementary Materials

The following supporting information can be downloaded at https://www.mdpi.com/article/10.3390/jcm15218713/s1, Table S1: Complete electronic search strategies used for the updated systematic review; Table S2: Reports assessed for eligibility at full text and excluded from the systematic review, with reasons for exclusion; Table S3: Detailed methodological quality assessment of the included studies using the Newcastle–Ottawa Scale (NOS); Supplementary Table S4: PRISMA 2020 Checklist.

## Abbreviations

ATA	Atmospheres Absolute
CI	Confidence Interval
FET	Frozen Embryo Transfer
GRADE	Grading of Recommendations Assessment, Development and Evaluation
HBOT	Hyperbaric Oxygen Therapy
HBO	Hyperbaric Oxygen
HIF-1α	Hypoxia-Inducible Factor 1 Alpha
ICSI	Intracytoplasmic Sperm Injection
IVF	In Vitro Fertilization
MD	Mean Difference
NOS	Newcastle–Ottawa Scale
POR	Poor Ovarian Response
PRISMA	Preferred Reporting Items for Systematic Reviews and Meta-Analyses
RR	Risk Ratio
SIRT1	Sirtuin 1
SIRT3	Sirtuin 3
WHO	World Health Organization
ART	Assisted Reproductive Technology
AMH	Anti-Müllerian Hormone
AFC	Antral Follicle Count
GEE	Generalized Estimating Equation
