# Supplementary material for: Hyperbaric Oxygen Therapy and Female Infertility: A Systematic Review of Human Clinical Evidence
Source: J Clin Med. 2026 Nov 8;15(21):8713. doi: 10.3390/jcm15218713 (PMC13764405; doi:10.3390/jcm15218713)
Supplement: Supplementary file 1 [file jcm-15-08713-s001.zip › jcm-4602257-Tables S1-S3.pdf]

Supplementary Table S1. Complete electronic search strategies used for the updated systematic review.

**Database**	**Search strategy**	**Date limits**	**Records identified**
**PubMed/MEDLINE**	(“Infertility, Female”[Mesh] OR “female infertility”[tiab] OR infertility[tiab] OR “unexplained infertility”[tiab] OR “poor ovarian reserve”[tiab] OR “recurrent implantation failure”[tiab]) AND (“Hyperbaric Oxygenation”[Mesh] OR “hyperbaric oxygen therapy”[tiab] OR “hyperbaric oxygen”[tiab] OR HBOT[tiab] OR HBO[tiab])	January 1, 2016–May 10, 2026	**18**
**Scopus**	TITLE-ABS-KEY (“female infertility” OR infertility OR “unexplained infertility” OR “poor ovarian reserve” OR “recurrent implantation failure”) AND TITLE-ABS-KEY (“hyperbaric oxygen therapy” OR “hyperbaric oxygenation” OR “hyperbaric oxygen” OR HBOT OR HBO)	January 1, 2016–May 10, 2026	**54**
**LILACS**	(infertility OR “female infertility”) AND (“hyperbaric oxygen therapy” OR HBOT OR “hyperbaric oxygen”) AND instance:“lilacsplus”	January 1, 2016–May 10, 2026	**1**
**Cochrane Library**	(“female infertility” OR infertility OR “unexplained infertility” OR “poor ovarian reserve” OR “recurrent implantation failure”) AND (“hyperbaric oxygen therapy” OR “hyperbaric oxygenation” OR “hyperbaric oxygen” OR HBOT OR HBO) in Title Abstract Keyword	January 1, 2016–May 10, 2026	**3**
**Total**	—	—	**76**

Supplementary Table S2. Reports assessed for eligibility at full text and excluded from the systematic review, with reasons for exclusion. Of the 11 reports assessed at full text, nine were excluded and two met the eligibility criteria and were included in the systematic review.

Study/report	Reason for exclusion
**Çağlı et al., 2023**	Animal study: cyclophosphamide-induced premature ovarian insufficiency rat model.
**Li et al., 2024**	Animal study: mouse embryo development and hyperbaric oxygen exposure.
**Feng et al., 2021**	Review article; no original patient data.
**Tchirikov et al., 2018**	Ineligible population/outcome: placental insufficiency during established pregnancy rather than female infertility or an infertility-related treatment condition.
**Wang et al., 2022**	Review article; no original patient data.
**Karaman et al., 2025**	Animal study: ovarian torsion model.
**Ma et al., 2023**	Animal study: aged mouse model.
**Syahrizal et al., 2022**	Animal study: endometriosis mouse model.
**Book Chapter – Textbook of Hyberbaric Medicine**	No original patient data.

Supplementary Table S3. Detailed methodological quality assessment of the included studies using the Newcastle–Ottawa Scale (NOS). The Newcastle–Ottawa Scale evaluates non-randomized cohort studies across three domains: Selection, Comparability, and Outcome. A maximum of nine stars can be awarded. Studies scoring 7–9 stars were classified as high quality, those scoring 4–6 stars as moderate quality, and those scoring 0–3 stars as low quality.

NOS domain/item	Chen et al., 2023	Meng et al., 2025
**Selection**		
Representativeness of the exposed cohort	★	★
Selection of the non-exposed cohort	★	—
Ascertainment of exposure	★	★
Demonstration that outcome of interest was not present at start of study	★	★
**Comparability**		
Comparability of cohorts on the basis of design or analysis	★★	★
**Outcome**		
Assessment of outcome	★	★
Was follow-up long enough for outcomes to occur?	★	★
Adequacy of follow-up of cohorts	★	★
**Total NOS score**	**9/9**	**7/9**

NOS total scores are reported descriptively and should not be interpreted as indicating absence of important risk-of-bias concerns. For Meng et al., the single comparability star reflects the study's adjusted analysis and does not represent an independent concurrent control group.
